# Adverse reactions to the use of large language models in social interactions

**DOI:** 10.1093/pnasnexus/pgaf112

**Published:** 2025-04-07

**Authors:** Fabian Dvorak, Regina Stumpf, Sebastian Fehrler, Urs Fischbacher

**Affiliations:** Centre for the Advanced Study of Collective Behaviour, University of Konstanz, Konstanz 78464, Germany; Department of Environmental Social Sciences, Eawag, Dübendorf 8600, Switzerland; Department of Economics, University of Konstanz, Konstanz 78464, Germany; TWI, Thurgau Institute of Economics, Kreuzlingen 8280, Switzerland; SOCIUM, University of Bremen, Bremen 28359, Germany; Department of Economics, University of Konstanz, Konstanz 78464, Germany; TWI, Thurgau Institute of Economics, Kreuzlingen 8280, Switzerland; CESifo, Munich 81679, Germany

**Keywords:** large language model, AI, efficiency, economic games, ChatGPT

## Abstract

Large language models (LLMs) are poised to reshape the way individuals communicate and interact. While this form of AI has the potential to efficiently make many human decisions, there is limited understanding of how individuals will respond to its use in social interactions. In particular, it remains unclear how individuals interact with LLMs when the interaction has consequences for other people. Here, we report the results of a large-scale, preregistered online experiment (n=3,552) showing that human players’ fairness, trust, trustworthiness, cooperation, and coordination in economic two-player games decrease when the decision of the interaction partner is taken over by ChatGPT. On the contrary, we observe no adverse reactions when individuals are uncertain whether they are interacting with a human or a LLM. At the same time, participants often delegate decisions to the LLM, especially when the model’s involvement is not disclosed, and individuals have difficulty distinguishing between decisions made by humans and those made by AI.

**Significance Statement**

Large language models (LLMs), such as ChatGPT, are now widely integrated into everyday life and are often used to facilitate social interactions. However, human responses to the use of LLMs in social interactions are not well understood. We focus on situations where an interaction between two people is mediated by ChatGPT. People show reduced fairness, trust, trustworthiness, cooperation, and coordination when ChatGPT takes over the decision of the interaction partner. At the same time, we find that people often delegate decisions to the AI, while distinguishing between human and AI decisions is only possible when behavior is complemented by written justifications. Our findings underscore the need for measures to address widespread skepticism about the use of LLMs in social interactions.

## Introduction

Fairness, reciprocity, trust, cooperation, and coordination are fundamental to human welfare by promoting socially desirable outcomes and efficiency. Recent advances in the development of large language models (LLMs) have shown the potential to fundamentally change many aspects of life in modern societies, including these facets of human behavior. While it is clear that AI in general, and LLMs in particular, have tremendous potential to improve human lives (1–5) and increase economic productivity (6, 7), little is known about how humans respond to their use in social interactions. This is the focus of our study.

Human social behavior depends not only on the expected consequences of our actions for others (8, 9) but also on our beliefs about the behavior of others (10), which together explain the variability of culture and social norms (11). When decisions in social interaction are delegated to LLMs, individuals’ beliefs and preconceptions about the nature of these models and the circumstances under which they are deployed become relevant factors influencing human decisions (12).

The primary focus of this study is *not* how LLMs will behave in social interactions, which has been studied in a recent string of other papers (13–17). Instead, we examine human reactions to the use of LLMs in social interactions, an aspect that has been underexplored. Research on human–algorithm interaction has shown that, depending on the design of the algorithm, humans often act more rationally and selfish than in human–human interactions, and seem less prone to emotional and social responses (18–20) but are nevertheless willing to delegate decisions to algorithms (21). In contrast to algorithms that have been optimized for specific tasks, LLMs provide an almost universal tool for social interaction in the digital age, where most social interactions are text-based, and LLMs are capable of producing adequate social judgments from written statements (22), or providing personalized recommendations based on individual preferences (23).

A key feature of our study is that the LLM acts on behalf of a real participant who is affected by the consequences of the interaction, which is relevant for humans with social preferences (24).

For this realistic scenario, several fundamental questions about human behavior in social interactions mediated by LLMs remain unanswered. What are the payoff consequences of human responses to the use of LLMs in social interactions? Under what circumstances are people willing to delegate decisions to LLMs? What role does the transparency of AI decisions play? And does the potential to personalize LLMs influence how people react to their use?

We conduct a preregistered online experiment (n=3,552) to investigate the effects of incorporating an LLM into human social interactions. In the main experiment, 2,905 participants (mean age: 39.9 years, 49.6% female, 47% college or university degree) engage in direct interactions either with other participants or with the LLM ChatGPT (25) acting on behalf of a human participant with AI support. In each interaction, one of the two participants is supported by ChatGPT. Both participants are affected by the consequences of the interaction, even though the AI is acting on behalf of the AI-assisted participant. We give the human participants and the AI the same instructions and ask for their decisions along with short justifications in five standard two-player games: the Ultimatum Game (UG) (26), the binary Trust Game (bTG) (27), the Prisoner’s Dilemma Game (PD) (28), the Stag Hunt Game (SH), and the Coordination Game (C). For all five games, there is a large body of empirical research documenting how people interact under controlled laboratory conditions (29–32). To incentivize decisions, each pair of participants receives the payoffs resulting from a randomly selected interaction.

The experiment uses a between-subjects design with six treatments. In the first treatment condition (*transparent random*), the AI randomly decides on behalf of the participant with AI support with a probability of 50%. Participants without AI support make two decisions—one for the case of interacting with a human and another for interacting with AI—making the use of AI transparent. In the second treatment condition, the participant with AI support has the option of delegating her decision to the AI (*transparent delegation*). Meanwhile, the participant without AI support makes two decisions, considering each possible outcome of the delegation decision. The third treatment condition is a variation of the delegation treatment in which the participant without AI support cannot condition her decision on the outcome of the delegation decision, making the use of AI opaque (*opaque delegation*). For each of these three treatment conditions, we vary whether participants can personalize the decisions the AI makes on their behalf at the beginning of the experiment (*personalized*) or not (*nonpersonalized*), resulting in a total of six between-subjects treatments.

To personalize the AI’s decision making, participants answered seven binary questions about their own personality before receiving the game instructions. The questions followed a simple “A or B?” format with the following pairs of alternatives: Intuition or Thoughtfulness, Introversion or Extraversion, Fairness or Efficiency, Chaos or Boredom, Selfishness or Altruism, Novelty or Reliability, and Truth or Harmony. Participants knew that their answers to the seven binary preference questions would be used to personalize the AI decisions made on their behalf. They also knew that whenever they interacted with the AI during the experiment, the algorithm would make decisions according to the other participant’s preferences, which were elicited through the same questions. We sampled personalized AI decisions for each possible combination of the seven binary preferences ($2^{7}=128$) by asking ChatGPT to generate decisions made by a person whose preferences were reflected in a particular response pattern (see Methods for details).

In addition to the main experiment, we test whether AI decisions can be detected in social interaction by showing sets of AI and human decisions to 647 human raters (mean age: 39.8 years, 50.1% female, 43.0% college or university degree). Raters receive a bonus payment if they correctly identify the decisions made by the AI. We also perform Turing tests by providing the human raters with written justifications for the decisions generated by the human and the LLM.

## Experimental results

To quantify the consequences of using LLMs in social interactions, we use preregistered behavioral indices (see Table 2 and Ref. (34)). To construct the indices, we sum the signed, normalized decisions in the games and use the average of the individual averages. Our payoff index is a summary of all decisions in terms of their impact on player’s payoffs. Three additional indices quantify the impact of prosociality, reciprocity, and beliefs about the kindness of the other player, and quantify their influence on the payoff effects.

The main hypothesis tests that we present in Table 1 were preregistered. Reported *P*-values are adjusted for multiple testing using the Holm–Bonferroni method (33). The main results are summarized in the same table, which describes the research questions and the corresponding answers derived from our experimental results for each question. The table indicates the variable tested and the treatment conditions compared.

**Table 1.** Key findings of the main experiment.

Research question		Variable	Cond. 1	Cond. 2	*P*-val
1. Does interacting with AI decrease payoffs?	Yes	Payoff index	HU: 0.69	AI: 0.65	<0.001
2. Is opaque delegation more frequent?	Yes	Delegation freq.	TD: 0.38	OD: 0.41	0.010
3. Does opaque interaction decrease payoffs?	No	Payoff index	HU: 0.69	UN: 0.71	1.000
4. Does delegation crowd-out prosociality?	No	Prosociality index	R: 0.55	D: 0.53	0.253
Does personalizing the AI…					
5. …restore payoffs?	No	Payoff index	P: 0.66	NP: 0.65	0.966
6. …increase delegation?	No	Delegation freq.	P: 0.39	NP: 0.40	1.000
7. …change payoffs if delegation is opaque?	No	Payoff index	P: 0.70	NP: 0.71	1.000
8. …change prosocial behavior?	No	Prosociality index	P: 0.55	NP: 0.53	0.259

All hypothesis tests and indices were preregistered and controlled for multiple testing (https://osf.io/fb7jd/). Results of one-sided t tests with Holm–Bonferroni corrected *P*-values (33). Corrected *P*-values can be equal to 1. The alpha level used to define statistical significance is 5%. HU, decisions directed at human participant; AI, decisions directed at AI; UN, unknown interaction partner (AI or human); TD, transparent delegation; OD, opaque delegation; R, random takeover by AI; D, delegation; P, personalized AI; NP, nonpersonalized AI.

The key result of our study is that engagement with an LLM triggers adverse human reactions that lead to a decrease in payoffs (question 1). This result emerges consistently across all five experimental games, with significant consequences for participants’ payoffs. We also find that participants often delegate their decisions to the LLM, especially when the delegation is opaque (question 2). Surprisingly, we find that participants do not change their behavior when delegation is opaque (question 3), despite their expectation that others will delegate. Delegation does not crowd out the social preferences of individuals who are aware that the other person has delegated to the AI (question 4). We find that personalizing the AI model does not affect how people respond to it (questions 5–8). In particular, it does not restore the payoff loss caused by interacting with the AI. We will now address each question in more detail. First, we present the results from the nonpersonalized treatments, and then we highlight the effects of personalization.

### Does interacting with AI decrease payoffs?

When interacting with the AI, participants’ decisions lead to a significantly lower value of our payoff index (M=0.65, SD = 0.19) than when interacting directly with a human (M=0.69, SD = 0.17, t(484) =−5.54, P<0.001). This is also true when the other person has deliberately delegated the decision to the AI (AI: M=0.63, SD = 0.19, HU: M=0.71, SD = 0.17, t(491) =−10.49, P<0.001) and when the AI is personalized to reflect the other person’s preferences (AI: M=0.66, SD = 0.19, Human: M=0.71, SD = 0.18, t(485) =−6.62, P<0.001). In accordance with the preregistration, we analyze which components of the payoff index contribute to this result. Belief in the kindness of the other player is significantly reduced when interacting with AI (M=0.61, SD = 0.32) compared to interacting directly with humans (M=0.66, SD = 0.31, t(484) =−3.78 , P<0.001). The frequency of modal choice in the coordination game, which we use as a measure of the predictability of participants’ decisions, is significantly lower when interacting with AI (AI: M=0.57, SD = 0.50, HU: M=0.64, SD = 0.48, t(484) =−2.82, P=0.003). The normalized offer in the ultimatum game, which can be seen as an incentivized measure of equality concerns, is also significantly lower when interacting with AI (M=0.91, SD = 0.31) compared to direct interaction with a human (M=0.98, SD = 0.24, t(484) =−5.01, P<0.001). Reciprocity does not seem to play a role in explaining payoff losses when interacting with AI (intention index, AI: M=0.62, SD = 0.19, HU: M=0.63, SD = 0.19, t(484) =−1.40, P=0.082).

We use linear regression models to examine the extent to which the AI-induced payoff loss is explained by participants’ prior experience with ChatGPT, their socioeconomic characteristics, and their attitudes toward AI (models (4)–(6) in Table S1 in the Supplementary material). We find that prior experience with ChatGPT does not significantly reduce the payoff loss. Age and positive attitudes toward AI, such as the belief that AI is trustworthy and the concern for equality when interacting with AI, reduce payoff loss, while the subjective difficulty of predicting AI decisions increases it. Participants who took longer in the experiment show a higher payoff loss. The size of the AI-induced payoff effect is also robust to controlling for participants’ socio-economic characteristics, their prior use of ChatGPT, and their attitudes toward AI (see models (1)–(3) in Table S1).

### Is opaque delegation more frequent?

We find that the propensity to delegate the decision to the LLM increases when delegation is opaque. Comparing the frequency of delegation in the transparent delegation treatments (M=0.38, SD = 0.28) with the frequency of delegation in the opaque delegation treatments (M=0.41, SD = 0.29), we find that the propensity to delegate increases by 3 percentage points (t(1973) = 2.97, P=0.010). This could be explained by the fact that participants anticipate the negative payoff consequences when the use of AI is transparent and shy away from transparent delegation. In a postexperimental questionnaire, we asked participants to rate the appropriateness of delegation on a five-point Likert scale. Participants’ responses indicate that delegation is generally appropriate, but transparent delegation is considered less appropriate than opaque delegation (transparent delegation [0–4]: M=2.48, SD = 0.97, opaque delegation [0–4]: M=2.60, SD = 0.92, t(1920) =−2.92, P=0.004).

### Does opaque delegation decrease payoffs?

Unexpectedly, we find that payoffs are not affected when opaque delegation to the LLM is possible. The decisions of participants who do not know whether they are interacting with a human or a LLM produce a similar level of the payoff index (M=0.71, SD = 0.17) compared to the decisions of participants who know that they are interacting with a human (M=0.69, SD = 0.17, t(974) = 1.48, P=1.000). At the same time, participants often delegate their decisions to the LLM (M=0.41, SD = 0.29) when the delegation is opaque and also expect others to use opaque delegation according to a postexperimental question (belief in delegation [0–4]: M=2.86, SD = 0.84).

### Does delegation to AI crowd out prosocial behavior?

By comparing participants’ decisions when interacting with the LLM in situations where the decision was delegated to the LLM with situations where the LLM took over randomly, we can study participants’ responses to delegation. We find that delegation does not crowd out social preferences. Prosocial behavior is unchanged in situations where delegation took place (prosociality index: M=0.53, SD = 0.19) compared to situations where the LLM took the decision randomly (M=0.55, SD = 0.20, t(971) =−1.73, *P*  = 0.253). This finding is further supported by participants’ responses to a question in the postexperimental questionnaire, which indicated that transparent delegation was generally considered appropriate (appropriateness (0–4): M=2.48, SD = 0.97, compared to indifference (2), t(964) = 15.27, P<0.001).

### Does personalization of the AI matter?

We find that personalizing the LLM does not restore the payoff loss resulting from the use of AI in social interaction. The payoff consequences of participants’ decisions are similar in the nonpersonalized random treatment (M=0.65, SD = 0.19) and the personalized random treatment (M=0.66, SD = 0.19, t(968) = 0.70, P=0.966). This is also true for the opaque delegation treatments (nonpersonalized: M=0.71, SD = 0.17, personalized: M=0.70, SD = 0.18, t(1006) =−1.19, P=1.000). Personalization also does not increase the propensity to delegate to AI (nonpersonalized: M=0.40, SD = 0.29, personalized: M=0.39, SD = 0.29, t(1973) =−0.74, P=1.000). Responses to our postexperimental question indicate that the use of personalization has a notable positive effect on the perceived alignment between the AI’s decisions and those of the other individual, as indicated by participants’ responses (nonpersonalized [0–4]: M = 1.54, SD = 1.10, personalized [0–4]: M=1.94, SD = 1.12, t(2945) =−9.94, P<0.001). However, participants also indicate that the other person is not adequately represented by the personalized LLM (AI adequately represents human [0–4]: M=1.94, SD = 1.12, vs. indifference (2), t(1476) =−2.03, P=0.979) (see Fig. S2 for a comparison of human and AI behavior).

### Behavior in experimental games

**Fig. 1.**
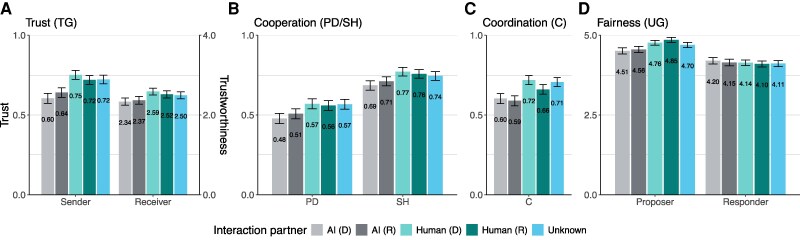
Average decisions in the Trust Game (A), the Cooperation Games (B), the Coordination Game (C), and the Ultimatum Game (D). From left to right, the bars represent decisions in which the interaction partner is AI in the transparent delegation treatments (first bar in each set), AI in the random treatments (second bar in each set), a human who does not delegate (third bar in each set), a human whose decision is implemented randomly (fourth bar in each set) or unknown (fifth bar in each set). Whiskers show bootstrapped 95% CI.

Figure 1 shows the average decisions in each game. We separate five cases: the case where the interaction partner is AI due to delegation (the first bar in each set), the case where the interaction partner is AI due to a random draw (the second bar in each set), the case where the interaction partner is a human who did not delegate (the third bar in each set), the case where the interaction partner is a human whose decision was implemented based on a random draw (the fourth bar in each set), and the case where the interaction partner is unknown (the last bar in each set). Whiskers show 95% CI obtained from nonparametric bootstrapping.

Comparing the AI-directed decisions with the human-directed decisions, we see that the AI induces payoff-decreasing decisions in all five experimental games. Pooling the AI-directed decisions (first two bars in each set) and the human-directed decisions (third and fourth bars in each set) for the delegation and random treatments, we observe the following significant differences. Figure 1A shows that participants place less trust in the LLM (M=0.62, SD = 0.48) than in humans (M=0.74, SD = 0.44, t(1935) =−9.91, P<0.001) and are less trustworthy when the trust decision was made by the LLM (M=2.35, SD = 1.50) than when it was made by a human (M=2.55, SD = 1.39, t(1935) =−8.72, P<0.001).

Figure 1B shows that participants cooperate less often in the Prisoner’s Dilemma when the other player’s decision is made by the LLM (M=0.49, SD = 0.50) than when it is made by a human (M=0.56, SD = 0.50, t(1935) =−6.32, P<0.001). The same result holds for the Stag Hunt Game (AI: M=0.70, SD = 0.46; HU: M=0.76, SD = 0.42, t(1935) =−6.72, P<0.001). Figure 1C shows that the predictability of participants’ actions in the coordination game suffers when interacting with the AI (M=0.60, SD = 0.49) compared to human interaction (M=0.69, SD = 0.46, t(1935) =−8.09, P<0.001).

Figure 1D shows that participants offer less as proposers in the Ultimatum Game when they know that the responder’s decision will be made by the LLM (M=4.53, SD = 1.56) compared to when the responder makes the decision (M=4.81, SD = 1.22, t(1935) =−9.64, P<0.001). When the LLM makes the offer in the Ultimatum Game, participants raise their minimum acceptance threshold and tolerate less negative inequality (M=4.17, SD = 1.62) than when the offer is made by a human (M=4.12, SD = 1.46, t(1935) = 2.22, P=0.027). This is the only situation in which the response to the LLM is potentially payoff increasing in the long run, as participants are willing to uphold the fairness norm at a personal cost. At the same time, the higher minimum acceptance threshold has negative consequences for efficiency, as it increases the probability of rejections.

The payoff-reducing choices made when interacting with the LLM are substantiated by participants’ responses in the postexperimental questionnaire. Participants find the AI less trustworthy and less cooperative than humans (trustworthiness and cooperation (0–4): AI: M=2.08, SD = 0.81; humans: M=2.25, SD = 0.71; test for difference in trustworthiness and cooperation (0): M=−0.17, SD = 0.99, t(2946) =−9.47, P<0.001), which explains less trust as a sender in the Trust Game and less cooperation in the Prisoner’s Dilemma and Stag Hunt Games when the other participant’s decision is made by the AI. Participants also indicate that they are less concerned about equality when AI makes the decision on behalf of the other person (equality concern (0–4): AI: M=2.99, SD = 1.05; human: M=3.26, SD = 0.94, test for difference in equality concern (0): M=−0.28, SD = 0.87, t(2946) =−17.25, P<0.001), which explains lower offers as proposers in the Ultimatum Game and lower back-transfers as receivers in the binary Trust Game.

The last bars in each set in Fig. 1 show that participants’ decisions in situations where they do not know whether they are interacting with a human or the AI, because delegation decisions are opaque, are similar to their decisions directed at humans who could have delegated their decision (fourth bars in each set). This is true for every decision in every game, suggesting that when in doubt, participants behave as if they were interacting with a human.

**Fig. 2.**
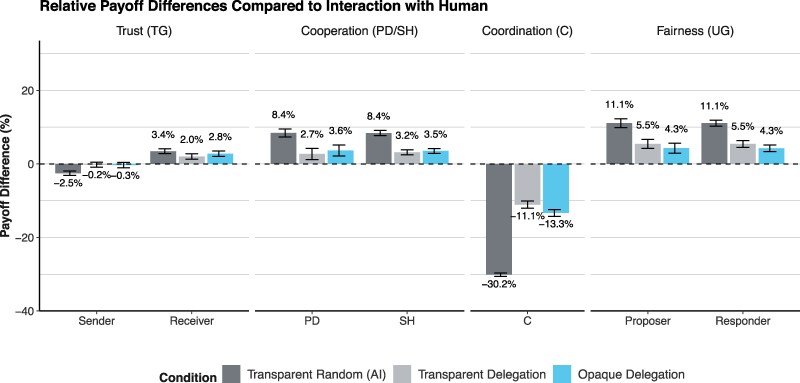
Differences in expected payoffs compared to the benchmark case where AI is not involved in the interaction. Average expected payoffs for participants with and without AI support. Whiskers show bootstrapped 95% CI.

Figure 2 shows the relative payoff differences for three cases: the case where a participant’s decision is taken over by the LLM in the transparent random treatment (transparent random [AI]), the case where a participant can transparently delegate to the AI (transparent delegation), and the case where a participant can secretly delegate to the AI (opaque delegation). We simulate the expected payoffs for all seven choices separately for the participant with and the participant without AI support by matching all participants of the same treatment, and compare the average expected payoff to the benchmark case where no AI is involved in the interaction (Fig. S3 in the Supplementary material) shows the differences in expected payoffs for the player with and the player without AI support separately). For the delegation cases, we implement the actual delegation decision of the participant who can delegate to the LLM.

The relative payoff differences shown in Fig. 2 indicate that the effect of AI-mediated social interaction on participants’ expected payoffs varies across games. The inclusion of AI increases the average expected payoffs in the Prisoner’s Dilemma and Stag Hunt Games, for the receiver in the Trust Game, and for both the proposer and responder in the Ultimatum Game. For the sender in the Trust Game, payoffs decrease only when the LLM randomly takes over the other participant’s decision, but not in the delegation treatments. In the Coordination Game, AI-mediated social interaction leads to significantly lower payoffs.

The relative payoff differences shown in Fig. 2 are mostly driven by the LLM’s decisions in the games (see Fig. S1). The decisions of the LLM are generally payoff increasing. For example, the AI always cooperates in the Prisoner’s Dilemma and Stag Hunt Games, which increases average payoffs and largely benefits the player without AI support (see Fig. S3). The AI also makes higher offers in the Ultimatum Game and accepts lower offers as a responder, which increases average payoffs. In contrast, the AI rarely chooses the payoff maximizing modal choice in the Coordination Game, which explains why the payoffs are significantly lower when the AI is involved. In the Trust Game, the AI often shows trust but little trustworthiness, which explains why the payoffs are shifted in favor of the receiver (see Fig. S3).

The relative payoff differences shown in Fig. 2 for the delegation cases, transparent delegation (second bar in each set) and opaque delegation (third bar in each set), are smaller compared to the first case because AI is only involved in the cases where decisions are actually delegated.

### Delegation behavior

**Fig. 3.**
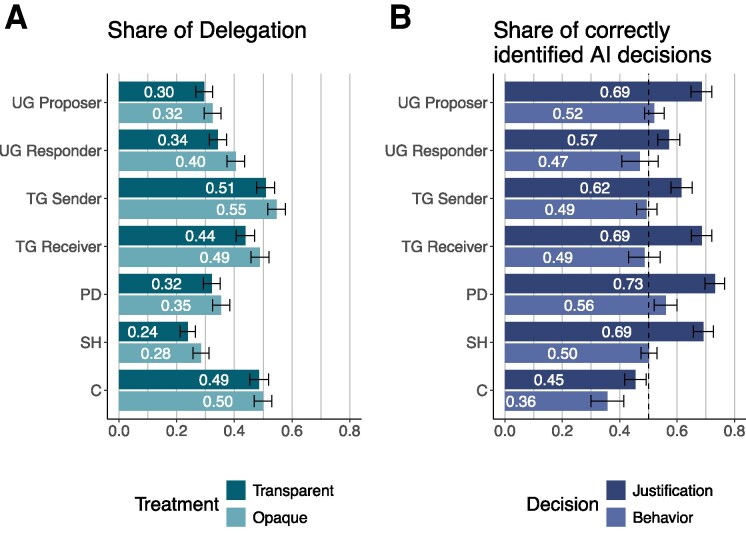
Delegation and detectability. Left: Share of delegated decisions in treatments *transparent delegation* (lighter bars) and *opaque delegation* (darker bars). Right: Share of correctly identified AI decisions of human raters when seeing pairs of AI and human decisions with (darker bars) and without statements of justification (lighter bars). Whiskers show bootstrapped 95% CI.

The left panel of Fig. 3 shows that at least 20% of all decisions in each game-specific situation are delegated to the AI. This is notable because the decisions have consequences. When participants delegate, they do not have to write a justification for the decisions, which creates an incentive to delegate. In addition, delegation can be used strategically to avoid being blamed for selfish decisions. Decisions in the Stag Hunt Game are delegated least often (transparent: 24%, opaque: 28%), while the binary trust decision is delegated over 50% of the time (transparent: 51%, opaque: 55%). This can be explained by the fact that cooperation seems to be very attractive in the Stag Hunt Game (M=0.81, SD = 0.39) and participants do not want to risk that the LLM does not cooperate. Contrasting the frequencies shown in the left panel of Fig. 3 with participants’ responses in the postexperimental questionnaire, we find that participants anticipate the actual delegation behavior by stating that others will more likely to delegate to the AI (belief in delegation (0–4): M=2.87, SD = 0.84, compared to neutrality (2), t(1975) = 45.96, P<0.001).

Figure 3 also shows that participants consistently delegate more if delegation is opaque (transparent delegation: M=0.38, SD = 0.28, opaque delegation: M=0.41, SD = 0.29, t(1973) = 2.97, P=0.010). In all games except the Coordination Game, opaque delegation is more frequent than transparent delegation (*P*-values in all situations except C < 0.081), which could indicate that participants anticipate the negative payoff consequences of transparent delegation.

### Detectability of delegation

We now turn to the question of whether humans are able to recognize AI decisions or AI-generated justifications for decisions. In each of the seven decisions, humans chose all available options with positive probability, making it fundamentally difficult to distinguish between human and AI decisions. The right panel of Fig. 3 shows that the 647 independent human raters who did not participate in the main experiment are generally unable to detect AI decisions. In all games except the Prisoner’s Dilemma, the success rate of human raters is not statistically better than random guessing (P>0.05 for all situations except PD) when presented with two decisions, one of which was generated by the LLM (lighter bars). In the Prisoner’s Dilemma, the LLM always cooperates, which is riskier, while humans sometimes choose to defect. This makes it possible to identify the AI (success rate PD: M=0.56, SD = 0.30, t(216) = 2.96, P=0.002). In contrast, the success rate of human evaluators in the Coordination Game is less than 50% (two-tailed t test: P<0.001), showing that humans can be misled by seemingly human-like AI decisions.

The darker bars in the right panel of Fig. 3 show that the AI generally fails the Turing test when the raters are additionally shown the written justifications for its decision. In all games except the Coordination Game, the success rate of human raters is significantly higher than chance (*P*-values for all situations except C < 0.001). The AI-generated justifications are generally more technical, often referring to the expected payoffs of the alternatives, than human justifications, which justify decisions in simple language. This may be due to the fact that the AI’s training data for the games comes mainly from texts aimed at a scientific audience, and consequently seems to use the same level of technicality in its justifications. The only exception is the Coordination Game, for which human evaluators cannot detect AI-generated content even when they have access to written justifications. For the Coordination Game, the AI justifications are less technical and sometimes even a bit arbitrary.

## Discussion

Recent advances in generative AI have paved the way for the convenient delegation of an increasing number of decisions to LLMs in everyday social interactions. The results of our large-scale experiment suggest that this development will be accompanied by adverse human reactions. Negative responses to interacting with an LLM persist in all five canonical games we study, pointing out a general skepticism toward generative AI. At the same time, prior experience with ChatGPT does not mitigate the adverse reactions when interacting with the LLM. Nearly half of our participants report having used ChatGPT at least once prior to the study, which suggests that the adverse reactions we find are not driven by a lack of familiarity with the LLM in our sample. The proportion of users among our participants is in line with a recent study on the adoption rate of ChatGPT among a representative sample of 18,000 employees in Denmark (35), of which 41% report having used the tool for work.

Despite the negative reactions to the LLM in social interactions, participants often benefit from interacting with the AI and consistently choose to delegate their decisions to the large language model when given the opportunity. This is particularly noteworthy given the now widespread and free availability of sophisticated LLMs. Notably, participants are more likely to delegate their decisions when the use of AI is opaque. This suggests an expectation of potential negative consequences associated with transparent delegation.

In our study, personalizing the LLM has no effect on how participants interact with the AI. This could be due to the way we personalize the LLM. Compared to previous studies that let LLMs learn individual preferences based on past decisions (23), our way of personalizing the LLM involved prompt engineering. While participants believe that this way of personalizing the LLM works to some extent, they do not believe that the other person’s preferences are adequately represented by the personalized LLM.

In addition, we find that opaque delegation to an LLM can evade detection under certain conditions. In four of the five games we study, decisions made by a modern LLM are indistinguishable when only decisions are reported. In one game, the accuracy of human raters tasked with identifying decisions covertly delegated to AI is even below chance. These results underscore the high quality that modern LLMs have achieved in standardized social interactions such as those examined in our study. However, when raters are provided with justifications for each decision, either written by a human participant or by the LLM, they can identify the AI decisions more often than chance in four out of five games. This illustrates that, despite progress, some limitations remain in the covert use of LLMs in human interaction. While the LLM we study in this article is capable of making reasonable choices in social interactions, the model still struggles to capture the underlying reasons why people make these choices, which are often highly complex and vary between individuals. However, this may change in future versions, as the evolution of LLMs is highly dynamic.

Our results corroborate the well-established finding of algorithm aversion in humans (18, 20, 21, 24) but extend this finding beyond specialized algorithms to the use of LLMs in social interactions. While we cannot exactly pinpoint the mechanisms that lead to the adverse reactions to the use of LLMs, which may be specific to each of the seven different interactions we studied, a general lack of trust seems the most natural candidate for a preliminary explanation for these effects.

Taken together, our findings challenge the prevailing assumption, that transparency alone is sufficient to safeguard the use of AI in social interactions. They suggest that regulation to protect against potential negative societal impacts of AI may be inadequate if it does not take into account adverse human reactions to such technology. For example, while the EU AI Act, one of the world’s first comprehensive AI laws, requires transparency regarding content generated by generative AI, our findings suggest that transparency needs to be complemented by measures that increase public trust in algorithms, such as independent, rigorous testing. Otherwise, transparency may also have unintended consequences. Paradoxically, the proposition to enhance transparency in AI usage, often advocated to minimize negative effects of generative AI on society, results in the most pronounced negative effects on payoffs in our study (also see (36, 37)).

While our article provides an initial account for understanding how LLMs will affect social interactions, we would like to point out some limitations that provide avenues for future research. First, while our online sample is fairly representative of the United Kingdom in terms of age, gender, and educational background, it certainly lacks representativeness in other important dimensions. Understanding how individual socioeconomic characteristics mediate the adverse reactions to the use of LLMs in social interactions remains critical to assessing the generalizability of our findings. Second, it should be noted that our results may not generalize to repeated interactions, where misconceptions about AI decisions in social interactions may be resolved over time, which should be addressed by future research. Third, it remains unclear how the way we personalize the LLM through prompt engineering compares to other methods of personalizing LLMs, which may yield different results. Finally, because our study cannot exactly identify the mechanisms driving the effects on payoffs, a more in-depth analysis of each type of social interaction, focusing on the underlying mechanisms, remains a crucial task for future studies.

## Methods

The study combines online data collection of AI decisions with an online experiment involving human participants. AI decisions and justification statements are elicited using the text interface of ChatGPT (25) (Version Jan 30, free research preview) prior to the online experiment. Participants of the online experiment are recruited via Prolific (www.prolific.com) and randomly assigned to one of the six between-subject treatments. Raters are recruited online via Prolific.

### AI decisions

We sampled AI decisions together with a statement of justification for all decisions of the experiment using the text interface of ChatGPT, Jan 30 Version (courtesy of OpenAI). The algorithm received the same instructions the participants receive during the experiment and therefore knows the consequences of the actions for both players (in experimental currency units [ECU]).

We sampled personalized AI decisions for each possible combination of seven binary preferences ($2^{7}\;$=128). The algorithm is instructed to act like a person who has preferences defined by a specific pattern of answers to the seven binary questions (see Personalization of AI section).

After the algorithm made the decision, we asked for a statement of justification with the sentence: “Please provide short, informal justification for the decision.” Before moving to the next decision, we deleted all prior conversations and restarted the text interface. In rare cases, in which the algorithm did not provide a valid answer or justification, we deleted the prior conversation, restarted the text interface, and repeated the procedure outlined above.

### Experimental games

Participants of the online experiment play five incentivized economic games. The payoffs are in ECU and are converted to British pounds for payment. Ultimatum Game (UG)

UG proposer: Endowment of 10 ECU. Offer (0–10 ECU)

UG responder: Minimum acceptable offer (0–10 ECU)

Binary Trust Game (bTG)

bTG sender: Endowment of 5 ECU. Binary decision to send 2 ECU (tripled).

bTG trustee: Endowment of 5 ECU. Return in case of trust (0–6 ECU)

Prisoner’s Dilemma Game (PD)

Both C, both get 5 ECU. Both D, both get 3 ECU.

You C and the other D, you get 1 ECU and the other gets 8 ECU.

You D and the other C, you get 8 ECU and the other gets 1 ECU.

Stag Hunt Game (SH)

Both C, both get 8 ECU. Both D, both get 4 ECU.

You C and the other D, you get 1 ECU and the other gets 5 ECU.

You D and the other C, you get 5 ECU and the other gets 1 ECU.

Coordination Game (C)

The five options are:

mercury, venus, earth, mars, saturn

Same option, 5 ECU. Different options, 2 ECU.

For the second-mover decisions in the Ultimatum Game and in the binary Trust Game, we use the strategy method and ask participants for the minimum acceptable offer and the conditional back-transfer in the case of trust. In total, there are seven different situations in the online experiment (UG sender, UG responder, bTG sender, bTG trustee, PD player, SH player, and C player).

Participants encounter each situation twice, first as the person with AI support and then as the person without AI support. In case of AI support, depending on the treatment, the participants either have to select a decision for the case that AI does not take over or they have to decide whether to delegate the decision to AI. Without AI support, depending on the treatment, the participants either have to select a decision for each potential and known interaction partner—AI or human—or select one decision for an unknown interaction partner.

### Experimental treatments

The experiment uses a between-subject design with six treatments. In the first treatment condition, AI makes the decision of the participant with AI support with 50% probability (*transparent random*). Participants without AI support make two decisions, one for the case of interacting with a human, and one for the case of interacting with AI—which makes the use of AI transparent. In the second treatment condition, the participant with AI support can choose if she wants to delegate her decision to AI (*transparent delegation*). In this condition, the use of AI is also transparent as the participant without AI support makes two decisions, one for each outcome of the delegation decision. The third treatment condition is a variant of the delegation treatment in which participants without AI support cannot condition their decision on the outcome of the delegation decision, which makes the use of AI opaque (*opaque delegation*). For each of these three treatment conditions, we vary whether participants can personalize the decisions AI makes on their behalf at the beginning of the experiment (*personalized*) or not (*nonpersonalized*).

This results in six between-subject treatments overall:

1. TRN: transparent random nonpersonalized
2. TRP: transparent random personalized
3. TDN: transparent delegation nonpersonalized
4. TDP: transparent delegation personalized
5. ODN: opaque delegation nonpersonalized
6. ODP: opaque delegation personalized

Each participant is randomly assigned to one of the six treatment conditions: TRN, TRP, TDN, TDP, ODN, and ODP. In the treatments with random takeover of AI (TRN, TRP), AI takes over the decision of the AI supported participant with 50% probability.

In the transparent treatments (TRN, TRP, TDN, TDP), participants decide separately for the scenario of interacting with a human or the scenario of interacting with AI. In the opaque delegation treatments (ODN, ODP), participants are not aware of the delegation decision and therefore do not know if they interact with a human or with AI.

### Monetary incentives

After a participant has completed all 14 decisions, she is randomly matched to another participant from the same treatment. For each pair of participants, the experimenter randomly selects one of the 14 interactions, which determines the game and the players’ roles in the game, and randomly determines which participant is supported by AI. If the treatment involves the possibility of random takeover by AI (TRN, TRP), the experimenter randomly determines if AI makes the decision on behalf of the participant with AI support. The payoffs of both participants are then determined using the decisions of both players (human or AI) in the selected interaction. There is no feedback between interactions.

### Personalization of AI

In the treatments with personalization, we use participants’ answers to seven binary preference questions to personalize AI decisions made on their behalf, which results in 128 possible answer patterns. Each unique pattern of answers reflects a certain personality of the AI. The questions follow the simple format “A or B?” and are: intuition or thoughtfulness, introversion or extraversion, fairness, or efficiency, chaos or boredom, selfishness or altruism, novelty or reliability and, truth or harmony.

To communicate the preferences of this person to the algorithm, we use prompts with the following wording: “Pretend you are a person who prefers intuition over thoughtfulness, introversion over extraversion, fairness over efficiency, chaos over boredom, selfishness over altruism, novelty over reliability, and truth over harmony. Only answer the question at the end. Do NOT explain your answer.” For nonpersonalized decisions, we use the sentence: “Pretend you are a person. Only answer the question at the end. Do NOT explain your answer.” After the introductory sentence, the algorithm receives information about the game and the decision.

Participants know the purpose of the questions—that their answers will influence the decisions AI makes on their behalf in the online experiment. Participants do not know the instructions of the games at the time when answering the binary questions, which limits (but does not exclude) strategic personalization. At the time when participants personalize the AI, they only know that they will interact with other participants and that their decisions will have consequences for both participants.

### Experimental procedures

All participants receive general instructions with information about the study and the experimental procedures and are subsequently asked to give informed consent. Participants then receive detailed information about the AI model used in the online experiment. We provide examples of model output in tasks unrelated to the economic games studied in the experiment. We then ask for participants’ prior experience with ChatGPT. In the treatments with personalized AI, we provide an additional example for the effect of personalizing the response of ChatGPT. Participants are then asked seven binary questions in the simple format “A or B?” to personalize decisions made by AI on their behalf. This happens before participants receive the instruction for the games to limit the possibility of strategic personalization.

Participants receive general instructions on how the interaction with the AI model works (treatment specific but not game specific) and answer several control questions including two attention checks. Participants then receive the instructions for the first game. We use standardized instructions for all games (38, 39) with minor modifications for SH and C. Participants are informed about their role in the game and are asked for their decision(s) with AI support.

For all decisions except those that are delegated to AI, participants write a short statement of justification immediately after making the decision. Participants know that the justifications will not be shown to other participants during the experiment. Participants are subsequently asked for their decision(s) as participant without AI support and their statement(s) of justification. If the game has more than one player role, the procedure is repeated for the other role. No feedback about the decisions of other participants or the AI decisions is provided between the interactions.

Participants with AI support make one decision in the treatments with random AI takeover (TRN, TRP) for the case that AI does not make the decision. In all treatments with delegation (TDN, TDP, ODN, ODP), participants with AI support first decide whether they would like to delegate and are subsequently only asked for their decision if they do not delegate to AI. Participants in the transparent treatments (TRN, TRP, TDN, TDP) make two decisions in the interactions without AI support, one for the case of interacting with a human and, one for the case of interacting with AI. Participants in the opaque delegation treatments (ODN, ODP) make one unconditional decision in interactions without AI support.

After a participant has completed all games in a randomized order, we ask participants for their age, gender, and education level. We also ask each participant to rate the predictability and kindness of AI, the importance of equality and intentions when interacting with AI, the resemblance of AI decision to human preferences, and in the delegation treatments the belief in and the appropriateness of delegation to AI on a 5-point Likert scale. After the experiment, each participant is matched to another participant from the same treatment by the experimenter. The experimenter randomly selects one of the 14 interactions for each pair and the matched participants receive the payoffs resulting from this interaction in addition to a flat payment for participation.

### Detectability and Turing tests

For each treatment condition, we create several collections of decisions, each consisting of seven human decisions and seven AI decisions. To generate the collections, we filter out all decisions with statements revealing that (i) a human made the decision or (ii) AI made the decision. This includes, for example, all human decisions with statements that reference information not available to AI (e.g. information about the general procedure of the experiment, the fact that the participant is interacting with AI) and AI statements revealing AI-generated decisions. We also filter out human statements with <3 words and correct the selected human statements for typos or grammatical errors.

For the data of four treatments TRN, TRP, TDN, and TDP, each rater compares the seven decisions a participant made without AI support targeted at a human to the seven AI decisions generated for the same participant in the same interaction and indicates for each situation which decision was generated by AI. All decisions are presented together with the corresponding statements of justification written by the participant or written by AI. The rater receives a bonus payment if her rating in one randomly selected situation is correct.

For the data of the treatments ODN and ODP, the raters compare decisions of participants with AI support who do not delegate their decision to the decision generated by AI for these participants in the same situation. We collect 14 ratings from each rater. Each rater reviews seven decisions, one from each of the seven situations in the online experiment. First, we present a pair of decisions without the written statement of justification and ask the rater to decide which decision AI made. Then, we reveal the written statements of justification and give the rater the opportunity to revise her rating. At the end, one situation and one of the two ratings is randomly selected and the rater receives a bonus payment if the rating is correct.

For the Turing tests (exploratory hypotheses H9 and H10), we are interested in the share of correctly identified AI decisions. In the treatments ODN and ODP, we post hoc randomize the positioning of the AI decisions in situations in which the AI and the human actions were identical to exclude any ordering bias. We do so by setting the outcome variable of correctly identifying the AI decision to 0.5. We also analyze participants’ Likert-scale ratings about the nature of human–AI interaction and socio-demographic variables collected after the experiment. We also measure response times and how often a participant leaves or switches tabs in the browser (or cannot see the browser window) during the online experiment.

### Ethical approval and informed consent

The online experiment was carried out in accordance with the regulations of the ethics committee of the University of Konstanz. Informed consent was obtained from all participants prior to participation. All experimental protocols were approved by the institutional review board of Gesellschaft für Experimentelle Wirtschaftsforschung (GfEW). Institutional Review Board Certificate No. Ja89kRho (https://gfew.de/ethik/Ja89kRho).

### Sample size

The target sample size was 3,000 experimental subjects in the main experiment (500 per treatment) and 600 human raters (100 per treatment) for the Turing tests. After data exclusion (see below), we reached a sample size of 2,947 experimental subjects (mean age: 39.9 years, 49.3% women, 49.8% men, 0.7% nonbinary, 70.4% college or university degree) and 655 human raters (mean age: 39.8 years, 50.1% women, 49.0% men, 0.5% nonbinary, 71.0% college or university degree). We estimated the required sample size of each treatment based on a simulation. For each of our main hypotheses (Q1–Q8), we simulated data with effects of 5 ppt in the direction of the alternative hypothesis for each decision affected by the alternative hypothesis. Rejecting these hypotheses with probability ≥0.90 at an alpha level of 5%, using Holm–Bonferroni corrected *P*-values for multiple testing, required a sample size of ∼500 participants per treatment. As a technical check, we ran a pilot experiment online with 60 participants recruited via Prolific.

The median duration time in the first experiment was 17.0 min and participants earned 7 pounds per hour on average. In the second experiment, the median duration time was 8.6 min with average earnings of 14 pounds per hour.

### Data exclusion

We discard the observations of participants who report that they have been repeatedly disturbed and therefore recommend not to use their data in the analysis in an open question asked at the end of the experiment. Participants are informed that not using their data has no payoff consequences for them. We also discard the observation of participants who state in an open question at the end that they consulted ChatGPT for advice during the experiment. We also discard all decisions with unreasonable response times (either too fast or too slow). A decision is discarded if the logarithm of the time needed to make the decision is more than 3 SD away from the mean.

### Experimental variables

We observe participants’ decisions in 14 different interactions with another unknown participant from the same treatment. Our key variables of interest are the decisions participants make in the 5 two-player games, which are: the offer in UG, the minimum acceptance threshold in UG, the binary trust decision in bTG, the conditional back-transfer in case of trust in bTG, the binary cooperation decision in PD, the binary cooperation decision in SH, and the selection of the modal choice in C (earth).

We generate the following normalized variables:

- Normalized offer in UG = offer/5.
  Offers are usually between 1 and 5, offers larger than 5 are truncated.
- Normalized min acceptance threshold in UG = min/5.
  Thresholds are usually between 1 and 5, thresholds larger than 5 are truncated.
- Normalized back-transfer in bTG = back-transfer/6.
  Back-transfers are between 0 and 6 (no truncation needed).

Using the normalized variables, we generate four preregistered behavioral indices outlined in Table 2.

**Table 2.** Behavioral indices.

Index	Description	Game decisions (effect)
Payoff index	Combines all decisions with direct or implicit payoff consequences.	Normalized offer in UG (+), normalized minimum acceptance threshold in UG (+), binary trust decision in bTG (+), normalized back-transfer in bTG (+), cooperation in PD (+), cooperation in SH (+), modal choice in C (+)
Prosociality index	Combines decisions in which social preferences play a role. Prosocial decisions are defined as decisions that increase the (expected) payoff of the other participant.	normalized offer in UG (+), normalized minimum acceptance threshold in UG (–), normalized binary trust decision in bTG (+), normalized back-transfer in bTG (+), cooperation in PD (+), cooperation in SH (+)
Kindness index	Quantifies the beliefs in the kindness of the other player.	Normalized binary trust decision in bTG (+), cooperation in PD (+), cooperation in SH (+).
Intentions index	Quantifies the role of intentions.	Normalized minimum acceptance threshold in UG (+), normalized back-transfer in bTG (+).

Behavioral indices created from the normalized game decisions. All indices were preregistered (https://osf.io/fb7jd). In the payoff index, all decisions enter positively. The only decision for which a positive relationship with player’s payoffs is debatable is the minimum acceptance threshold in the ultimatum game. Using a positive weight requires that the long-run gains from maintaining the social norm of utility outweigh the short-run efficiency losses from rejection. The payoff results are robust to using a negative weight for the responder’s decision.

### Statistical analyses

We use a preregistered conditional testing procedure to correct for multiple testing. First, we test the main hypotheses shown in Table 1 using Holm–Bonferroni corrected *P*-values, which controls for the fact that we initially perform eight tests using an alpha level of 5%. If a test is significant, because its corrected *P*-value is smaller than 5%, we perform conditional tests for the same data. For significant tests involving the payoff index (Q1, Q3, Q5, and Q7 in Table 1), we additionally test for differences in the predictability of behavior (frequency of modal choice in C), equality concerns (offers in UG), the kindness index, and the intentions index (four conditional tests).

For significant tests involving prosociality (Q4, Q8), we test for differences in the six normalized decisions underlying the prosociality index: the normalized offer in the ultimatum game (offer/5), the normalized minimum acceptance threshold (min/5), the binary trust decision, the normalized back-transfer in the Trust Game (back-transfer/6), and the binary cooperation decision in the Prisoner’s Dilemma and the binary cooperation decision in the Stag Hunt Game (six conditional tests). For significant tests involving delegation (Q2, Q6), we test if the frequency of delegation differs in each game-specific situation (seven conditional tests). Simulation results suggest that the conditional testing procedure effectively restricts the familywise error rate. The simulated probability of at least one Type I error is 4.5% for the main tests and 4.3% for the conditional tests (estimated based on 10,000 simulation runs).

### Preregistration

The study was preregistered. The preregistration files of the study can be found here: https://osf.io/fb7jd/

## Supplementary Material

pgaf112_Supplementary_Data

## Data Availability

Data, code, and materials used in the analysis are deposited in the Open Science Framework repository: https://osf.io/fvk2c/.

## Preprints

This manuscript was posted on a preprint: https://doi.org/10.48550/arXiv.2401.12773. The project was part of Regina Stumpf’s cumulative dissertation (40). So, this article contains some overlapping material with it.
