# Supplementary material for: Adverse reactions to the use of large language models in social interactions
Source: PNAS Nexus. 2025 Apr 7;4(4):pgaf112. doi: 10.1093/pnasnexus/pgaf112 (PMC11997303; doi:10.1093/pnasnexus/pgaf112)
Supplement: pgaf112_Supplementary_Data [file pgaf112_supplementary_data.pdf]

Supplementary material

Adverse reactions to the use of large language models in social interactions

Fabian Dvorak, Regina Stumpf, Sebastian Fehrler and Urs Fischbacher

PNAS Nexus

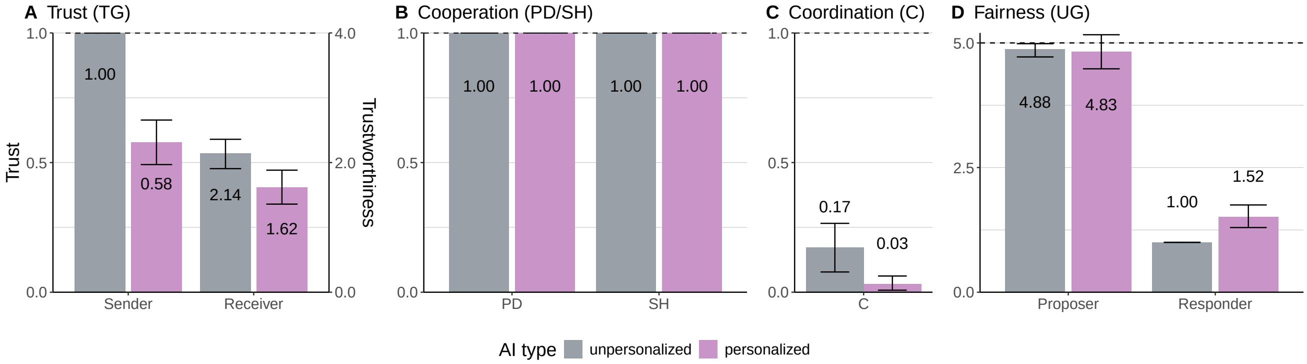

Fig. S1. AI decisions across four types of games: Trust Game (Panel A), Cooperation Games (Panel B), Coordination Game (Panel C), Fairness Game (Panel D). The color of the bars represent decisions of unpersonalized AI (gray bars), and personalized AI (pink bars). Whiskers show bootstrapped 95% confidence intervals.

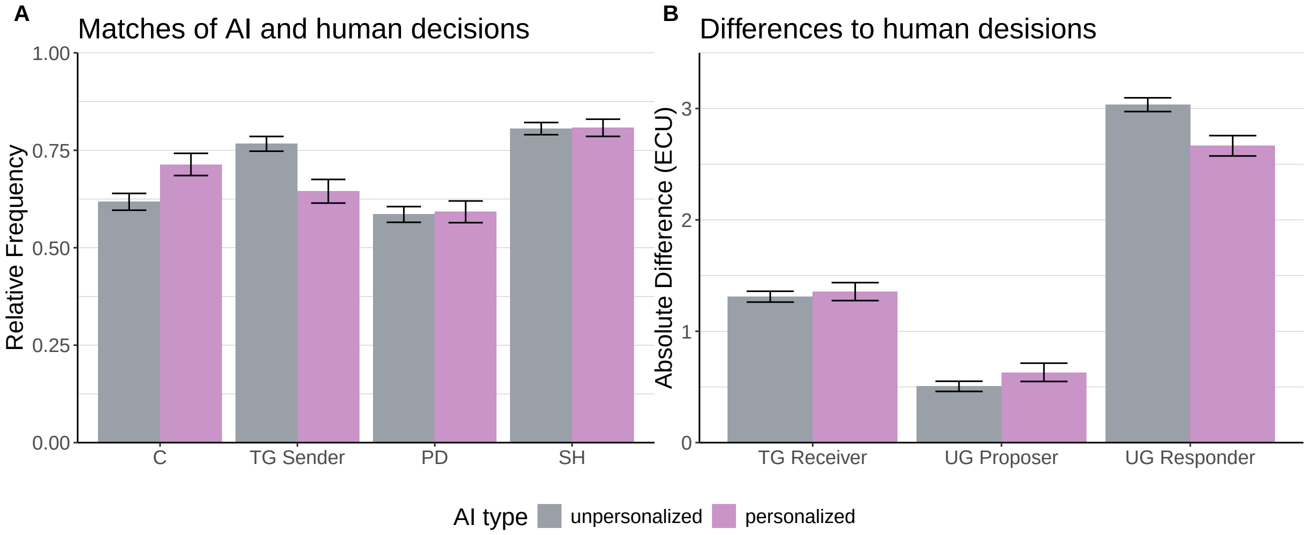

Fig. S2. Differences between AI and human decisions: Relative frequency of matches of binary decisions (Panel A) and absolute differences in non-binary decisions (Panel B). The color of the bars represent decisions of unpersonalized AI (gray bars), and personalized AI (pink bars). Whiskers show bootstrapped 95% confidence intervals.

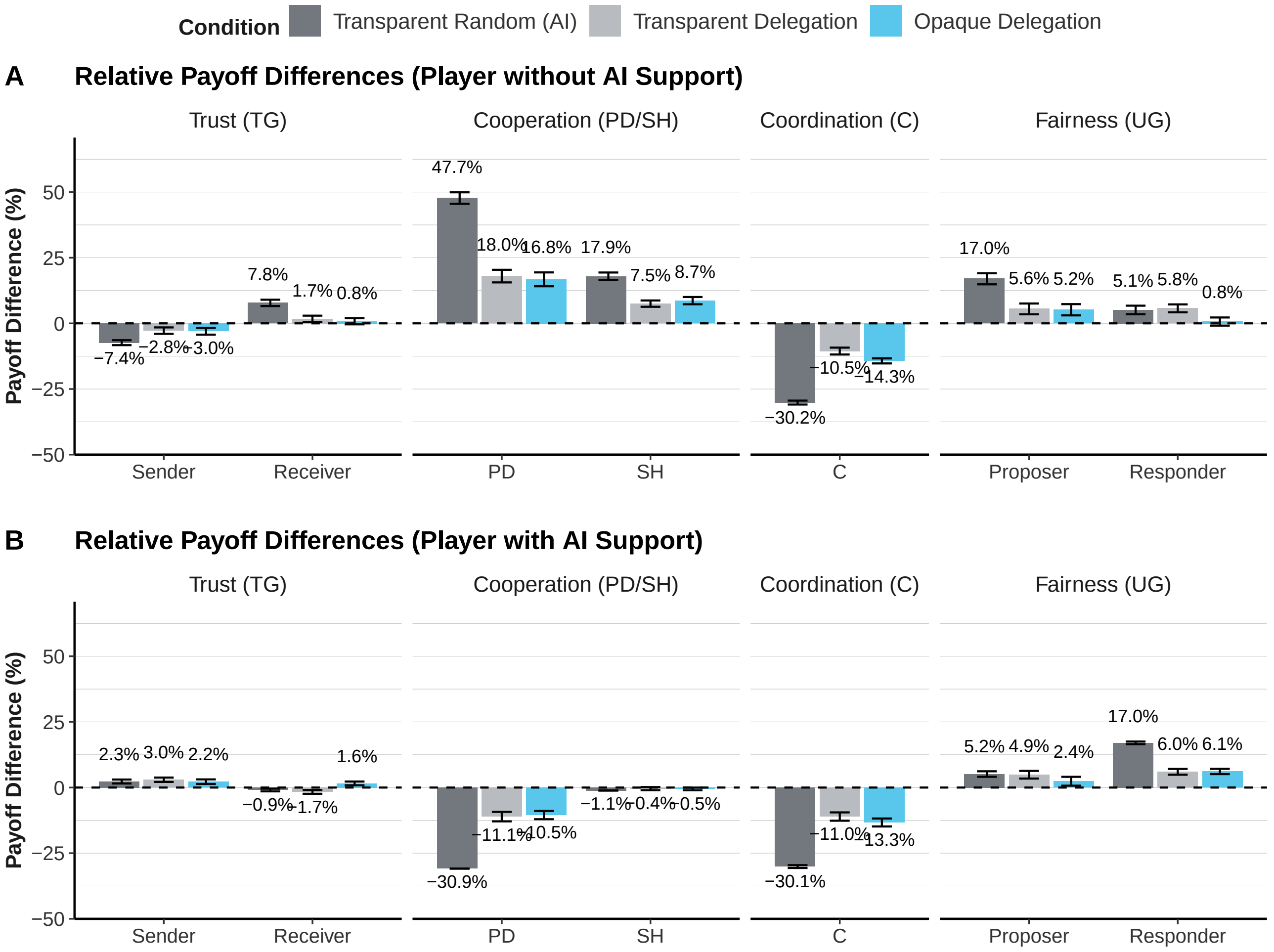

Fig. S3. Relative payoff difference in percent of the player without AI support (panel A), and the player with AI support (panel B) compared with human-human interactions in the games. Whiskers show bootstrapped 95% confidence intervals.

Table S1. Influence of covariates on payoff index

	Dependent variable:					
	Payoff Index (base: AI)			Diff. in Payoff Index (Human-AI)		
	(1)	(2)	(3)	(4)	(5)	(6)
Decision targeted at...						
...Human	0.060***	0.060***	0.060***			
	(0.004)	(0.004)	(0.004)			
...Unknown	0.054***	0.050***	0.046***			
	(0.007)	(0.007)	(0.007)			
Age		0.0003	0.0005**	−0.001	−0.0003	−0.001*
		(0.0002)	(0.0002)	(0.0003)	(0.0003)	(0.0003)
Gender: Non-binary		−0.036	−0.035	0.101**	0.084**	0.081*
		(0.033)	(0.034)	(0.043)	(0.042)	(0.042)
Gender: Woman		−0.013**	−0.013**	−0.001	−0.004	−0.004
		(0.006)	(0.006)	(0.008)	(0.008)	(0.008)
		(0.006)	(0.006)	(0.008)	(0.008)	(0.008)
Higher Education		−0.009	−0.009	−0.0001	0.0001	−0.0002
		(0.007)	(0.007)	(0.009)	(0.008)	(0.008)
Usage of ChatGPT		0.004	0.005	0.0004	0.002	0.002
		(0.003)	(0.003)	(0.004)	(0.004)	(0.004)
Agreement to...						
...AI difficult to predict		−0.006*	−0.006*		0.018***	0.017***
		(0.003)	(0.003)		(0.004)	(0.004)
...AI trustworthy		0.015***	0.015***		−0.036***	−0.037***
		(0.004)	(0.004)		(0.005)	(0.005)
...AI equality concerned		0.054***	0.054***		−0.015***	−0.015***
		(0.003)	(0.003)		(0.004)	(0.004)
...AI reflects human		−0.002	−0.002		−0.001	−0.001
		(0.003)	(0.003)		(0.003)	(0.003)
Distractions			−0.00004			−0.001*
			(0.0005)			(0.001)
Duration (min)			−0.001***			0.001***
			(0.0003)			(0.0004)
Constant	0.649***	0.476***	0.490***	0.080***	0.137***	0.123***
	(0.004)	(0.019)	(0.019)	(0.016)	(0.024)	(0.024)
Observations	4,883	4,883	4,883	1,936	1,936	1,936
R^2	0.024	0.137	0.139	0.005	0.063	0.069
Adjusted R^2	0.024	0.135	0.137	0.002	0.058	0.064

Note: OLS regressions with payoff index (baseline: decisions targeted at AI) observed in all treatments as dependent variable in models (1)–(3) and difference in payoff index (human - AI) observed in the treatments *transparent random* as dependent variable in models (4)–(6). *Usage of ChatGPT* is the numeric representation of the frequency of the use of ChatGPT. The four variables of agreement to specific statements about the AI are measured on a scale from 0–4. *Agreement to...* indicated on a five-point Likert scale ranging from completely disagree to completely agree. *Distractions* is the frequency of changing the browser window during the experiment. Robust standard errors clustered at the subject level in models (1)–(3). *p<0.1; **p<0.05; ***p<0.01.

Instructions Main Experiment

<u>Welcome!</u>

You are asked to participate in this study conducted by Fabian Dvorak, Urs Fischbacher, Regina Stumpf (University of Konstanz) and Sebastian Fehrler (University of Bremen).

<u>Important Information</u>

What to expect: This experiment consists of 7 situations in which you have to make decisions.

Voluntary participation: Participating in this study is completely voluntary. You can quit the study at any time. When quitting the study you will not be paid on the Prolific platform.

Anonymity: The study is anonymous and no one will be able to link your answers back to you. Please do not include your name or other information that could be used to identify you in the responses. Your responses will be used for research purposes only.

Confidentiality: The data collected in this study will be stored anonymously on encrypted servers of the University of Bremen and the University of Konstanz. The consent to the collection and processing of the data is irrevocable, as no participant-related deletion can be carried out due to the anonymized form of the study.

Have you carefully read and understood the above information and would like to participate in this study?
[Yes/No]

<u>INSTRUCTIONS (1 OF 3)</u>

This experiment consists of **7 situations,** in which you have to make decisions.

Each situation is about an **exchange between yourself and another person** with whom you are randomly matched.
You and the other person can earn money in a currency called ECU. The exchange rate is 1 ECU = 0.5 Pounds.
You do not know this other person and you will not be informed about his/her identity.

In the end, **one of the 7 situations** will be randomly chosen and you and the other person will be paid according to the decisions you made in this situation.
Therefore, you should decide in each situation like you would if it was selected for sure. You will not get any feedback about the decisions after each situation.
In addition to the bonus payment, you will receive **2 Pounds for completing the study.**
[OK]

<u>INSTRUCTIONS (2 OF 3)</u>

In each situation, one person will be **supported by artificial intelligence (AI).** This means that either you **or** the other person will be supported by AI, but never both of you.

The AI used in this experiment is the chatbot **ChatGPT** developed by OpenAI. ChatGPT is a machine learning model that uses transcripts of spoken language as well as written text from online forums, social media, newspaper articles and books to generate answers in a conversational way using a dialog format. Among other things, it has been shown that ChatGPT can debug computer programs; write poems, song lyrics, fairy tales, and student essays; answer test questions and play games.

Here is an example of an answer generated by ChatGPT:

Question:
"What is Prolific?"

Answer ChatGPT :
"Prolific is a platform that connects researchers with a large pool of participants for their studies. Researchers can use Prolific to design and run studies, and participants can sign up to take part in studies and earn money for their time. Prolific streamlines the recruitment process and makes it easier for researchers to find participants for their studies, while also providing a convenient way for participants to earn extra income by taking part in research."

For this experiment, **ChatGPT (Version Jan 30)** received the **same instructions** as you will for the specific decision situations.

Have you already heard of ChatGPT prior to this study?
[Yes/No]

How often have you used ChatGPT so far?
[Never/1-5 times/6-10 times/11-50 times/Over 50 times]

<u>INSTRUCTIONS (3 OF 3)</u>

In each situation of the main experiment there are two scenarios:

Scenario 1: You have AI support. This means that either you decide or the AI decides for you. The other person does not have AI support.

Scenario 2: The other person has AI support. This means that either the other person decides or the AI decides for the other person. You do not have AI support.

In each situation, you will make a decision for both scenarios. In the end, it will be randomly determined which scenario applies.

<u>Consequences of AI support:</u>

The person who is supported by the AI will either make the **decision on her own or delegate the decision to the AI.** The AI will then **decide on behalf of the person**.
The **AI receives the same instructions** for the specific situation before making a decision.

The **AI cannot earn any ECU** and the **consequences of the exchange will affect the person the AI is supporting.**

In each situation, the person **without AI support will always make the decision on her own.**
It will NOT be clear to neither of you, whether the person with AI support makes the decision on her own or whether she delegates the decision to the AI. This means if you are the person with AI support, then it will not be clear to the other person if you delegated the decision or not. If the other person is the person with AI support, then it will not be clear to you if the other person delegated the decision or not.

Whenever you make a decision on your own, you are asked for a short and informal justification of your decision. When you delegate a decision, you do not have to write a justification.
Important: These statements of justification will NOT be shown to any other person in this experiment.
[OK]

Before starting with the main experiment, please answer the following question concerning the instructions you just read.

You will have two opportunities to get this question correct.
Please select the correct statement.

In each situation, ...
[Both me and the other person will get AI support / Either me or the other person will get AI support / Neither me nor the other person will get AI support]

Before starting with the main experiment, please answer the following question concerning the instructions you just read.

You will have two opportunities to get this question correct.
Assume the AI will decide on behalf of the other person.

Who will be affected by the consequences of the AI's decision?

[Only the AI will be affected by the consequences/The other person and I will be affected by the consequences/The consequences do not matter as the AI decides]

We want to make sure that you are a real person and pay attention.
Please indicate your agreement with the following statement:

"I swim across the Atlatic Ocean to get to work every day."
[Likert Scale: strongly disagree – strongly agree]

<u>Situation 1 of 7</u>

The Transfer-Situation (Sender)

Situation 1 consists of an exchange between a **sender and a receiver.**
You have been assigned the role of the **sender**. The other person is in the role of the **receiver**.
You and the receiver are both endowed with 5 ECU.
You first decide if you want to **transfer 2 ECU** of your endowment to the receiver.
The amount you **transfer is tripled to 6 ECU** (by the experimenter) before being received by the receiver. The amount you keep is not tripled and simply remains in your possession.

The receiver then **decides how much of the 6 ECU to return to you.** The receiver can choose any amount between 0 ECU and 6 ECU. The amount the receiver returns is not tripled.
Your final payment is the sum of the amount of your 5 ECU endowment you keep plus the amount the receiver returns from your tripled transfer (in case you transfer 2 ECU).
The receiver's final payment is the sum of his/her 5 ECU endowment plus the amount the receiver keeps from your tripled transfer (in case you transfer 2 ECU).

Note that the receiver will not learn your transfer before making a decision on how much to return, but will indicate his/her return in the case of a transfer. After both of you have made your decisions, you and the receiver will receive the corresponding payment.

<u>Scenario 1:</u> **You have AI support**.

Do you want to make the decision on your own or delegate the decision to the AI?

Please note: It will NOT be clear to the other participant if you make the decision on your own or delegate the decision to the AI.

[Make decision on my own/Delegate decision to AI]

Do you want to transfer 2 ECU of your endowment to the receiver?
[Yes/No]

Please provide short, informal justification for the decision.
[Text]

<u>Scenario 2:</u> **The other participant has AI support.**

Please note: It will NOT be clear to you if the other participant makes the decision on her own or delegates the decision to the AI.

Do you want to transfer 2 ECU of your endowment to the receiver?
[Yes/No]

Please provide short, informal justification for the decision.
[Text]

Situation 2 of 7

The Transfer-Situation (Receiver)

Situation 2 consists again of an exchange between a **sender and a receiver**.
This time you have been assigned the role of the **receiver**. The other person is in the role of the **sender.**
You and the sender are both endowed with 5 ECU.
The sender first decides if he/she **transfers 2 ECU** of his/her endowment to you.

The amount the sender **transfers is tripled to 6 ECU** (by the experimenter) before being received by you. The amount the sender keeps is not tripled and simply remains in his/her possession.

You then decide **how much of the 6 ECU to return to the sender.** You can choose any amount between 0 ECU and 6 ECU. The amount you return is not tripled.
Your final payment is the sum of your 5 ECU endowment plus the amount you keep from the sender's tripled transfer (in case the sender transfers 2 ECU).
The sender's final payment is the sum of the amount of his/her 5 ECU endowment s/he keeps plus the amount you return from the sender's tripled transfer (in case the sender transfers 2 ECU).

After both of you have made your decisions, you and the sender will receive the corresponding payment.

Scenario 1: **You have AI support**.

Do you want to make the decision on your own or delegate the decision to the AI?

Please note: It will NOT be clear to the other participant if you make the decision on your own or delegate the decision to the AI.

[Make decision on my own/Delegate decision to AI]

How much of the 6 ECU do you return to the sender in the case of a transfer?
[Number]

Please provide short, informal justification for the decision.
[Text]

Scenario 2: **The other participant has AI support.**

Please note: It will NOT be clear to you if the other participant makes the decision on her own or delegates the decision to the AI.

How much of the 6 ECU do you return to the sender in the case of a transfer?
[Number]

Please provide short, informal justification for the decision.
[Text]

Situation 3 of 7

The Planet-Situation

You and the other are both asked to **choose between five options.** You choose independently of each other, that is, without knowing the other's choice.

Your own and the other's payment depend on the combination of your choices as follows:
If both of you choose the **same option you both get 5 ECU.**
If both of you choose **different options you both get 2 ECU**.

The five options are: **mercury, venus, earth, mars, saturn**

Scenario 1: **You have AI support**.

Do you want to make the decision on your own or delegate the decision to the AI?

Please note: It will NOT be clear to the other participant if you make the decision on your own or delegate the decision to the AI.

[Make decision on my own/Delegate decision to AI]

Which of the five options do you choose?
[mercury/venus/earth/mars/saturn]

Please provide short, informal justification for the decision.
[Text]

Scenario 2: **The other participant has AI support.**

Please note: It will NOT be clear to you if the other participant makes the decision on her own or delegates the decision to the AI.

Which of the five options do you choose?

[Mercury/Venus/Earth/Mars/Saturn]

Please provide short, informal justification for the decision.
[Text]

<u>Situation 4 of 7</u>

The Allocation-Situation (Proposer)

Situation 4 consists of an exchange between a **proposer and a responder**.
You have been assigned the role of the **proposer.** The other person is in the role of the **responder.**
You are endowed with 10 ECU, the responder is endowed with 0 ECU.
You are asked to **make a proposal on how to divide your 10 ECU** endowment between yourself and the responder. That is, you decide how much to transfer to the responder and how much to keep. This is your offer to the responder.
The responder then decides whether **to accept or reject this offer.**

Your and the responder's final payment depend on the responder's decision:
If the responder accepts, the **allocation is realized as proposed** in your offer. That is, you and the responder receive the amounts that you allocated to both of you.
If the responder rejects, neither you nor the responder receives any money; that is, **both of you get 0 ECU**.

Note that the responder will not learn your offer before making a decision on whether to accept or reject it, but will rather **indicate the minimum offer s/he would be willing to accept.**
If your offer falls below this minimum acceptance level, both of you receive nothing; if your offer is above or equal to the responder's minimum acceptance level, both of you receive the amounts as proposed by you.

<u>Scenario 1:</u> **You have AI support**.

Do you want to make the decision on your own or delegate the decision to the AI?

Please note: It will NOT be clear to the other participant if you make the decision on your own or delegate the decision to the AI.

[Make decision on my own/Delegate decision to AI]

How much of your 10 ECU endowment do you want to offer the responder?
[Number]

Please provide short, informal justification for the decision.
[Text]

<u>Scenario 2:</u> **The other participant has AI support.**

Please note: It will NOT be clear to you if the other participant makes the decision on her own or delegates the decision to the AI.

How much of your 10 ECU endowment do you want to offer the responder?
[Number]

Please provide short, informal justification for the decision.
[Text]

Situation 5 of 7

The Allocation-Situation (Responder)

Situation 5 consists again of an exchange between a **proposer and a responder.**
This time you have been assigned the role of the **responder.** The other person is in the role of the **proposer.**
The proposer is endowed with 10 ECU, you are endowed with 0 ECU.

The proposer makes a **proposal on how to divide his/her 10 ECU endowment** between him/herself and you. That is, the proposer decides how much to transfer to you and how much to keep.
This is the proposer's offer to you.

You decide **whether to accept or reject this offer.**
The proposer's and your final payment depend on your decision:
If you **accept**, the **allocation is realized as proposed** in the proposer's offer. That is, you and the proposer receive the amounts that the proposer allocated to both of you.
If you **reject**, neither you nor the proposer receives any money; that is, **both of you get 0 ECU.**

Please now think about the **minimum offer you would be willing to accept.** That is, how much of the 10 ECU endowment would the proposer need to offer you at minimum such that you would accept the offer. If the proposer's offer is below this minimum, you would reject it; if the proposer's offer is above or equal to this minimum, you would accept it.

Scenario 1: **You have AI support**.

Do you want to make the decision on your own or delegate the decision to the AI?

Please note: It will NOT be clear to the other participant if you make the decision on your own or delegate the decision to the AI.

[Make decision on my own/Delegate decision to AI]

What is the minimum offer in ECU you would accept?
[Number]

Please provide short, informal justification for the decision.
[Text]

Scenario 2: **The other participant has AI support.**

Please note: It will NOT be clear to you if the other participant makes the decision on her own or delegates the decision to the AI.

What is the minimum offer in ECU you would accept?
[Number]

Please provide short, informal justification for the decision.
[Text]

Situation 6 of 7

The A-B-Situation

You and the other are both asked to choose between **two options, A and B**. You choose independently of each other, that is, without knowing the other's choice.

Your own and the other's payment depend on the combination of your choices as follows:
If both of you choose A, you **both get 5 ECU**.
If you choose A and the other chooses B, **you get 1 ECU** and the **other gets 8 ECU**.
If you choose B and the other chooses A, **you get 8 ECU** and the **other gets 1 ECU.**
If both of you choose B, you **both get 3 ECU.**

Scenario 1: **You have AI support**.

Do you want to make the decision on your own or delegate the decision to the AI?

Please note: It will NOT be clear to the other participant if you make the decision on your own or delegate the decision to the AI.

[Make decision on my own/Delegate decision to AI]

Which option do you choose?
[A/B]

Please provide short, informal justification for the decision.
[Text]

Scenario 2: **The other participant has AI support.**

Please note: It will NOT be clear to you if the other participant makes the decision on her own or delegates the decision to the AI.

Which option do you choose?
[A/B]

Please provide short, informal justification for the decision.
[Text]

Situation 7 of 7

The X-Y-Situation

You and the other are both asked to **choose between two options, X and Y.** You choose independently of each other, that is, without knowing the other's choice.

Your own and the other's payment depend on the combination of your choices as follows:
If both of you choose X, you **both get 8 ECU**.
If you choose X and the other chooses Y, **you get 1 ECU** and the **other gets 5 ECU.**
If you choose Y and the other chooses X, **you get 5 ECU** and the **other gets 1 ECU.**
If both of you choose Y, you **both get 4 ECU.**

Scenario 1: **You have AI support**.

Do you want to make the decision on your own or delegate the decision to the AI?

Please note: It will NOT be clear to the other participant if you make the decision on your own or delegate the decision to the AI.

[Make decision on my own/Delegate decision to AI]

Which option do you choose?
[X/Y]

Please provide short, informal justification for the decision.
[Text]

Scenario 2: **The other participant has AI support.**

Please note: It will NOT be clear to you if the other participant makes the decision on her own or delegates the decision to the AI.

Which option do you choose?
[X/Y]

Please provide short, informal justification for the decision.
[Text]

You are almost done with the experiment. Please read the following statements and indicate how much you agree with them.
Afterwards, please click on continue.

The statements relate to the previous situations with AI interaction.
Think about the situations in which the other person had AI support and **in which you potentially had to interact with the AI.**

How much do you agree with the following statements?

[Beliefs and Attitudes towards AI, Likert Scale strongly disagree – strongly agree]

Please answer the following questions.
How old are you?
[Number]

Gender: How do you identify?
[Male/Female/Non-binary/Prefer to self-describe]

What is the highest level of education you have completed?
[Primary school/Secondary school/Higher education/Colleague or university/Post-graduate degree/Prefer not to say]

Have you consulted ChatGPT through other channels during this study to learn about the decisions of AI?

Note that your answer here has no payoff consequences for you and that you will get your payoff in any case.

[Text]

Have you been disturbed repeatedly during this study such that you wouldn't recommend using your answers in the analysis?

Note that your answer here has no payoff consequences for you and that you will get your payoff in any case.

[Text]

Do you have any feedback concerning the comprehension or feasibility of this study?
[Text]

Once the other participants are done with this experiment, you will be randomly matched to one of them. One situation and one scenario will be randomly determined for the both of you.

You will earn 2 Pounds for completing the experiment as well as the additional bonus payment generated from the decisions of the situation that has been selected for you.

Please click the button below to be redirected to Prolific and register your submission.

Instructions Turing Experiment

Welcome!

You are asked to participate in this study conducted by Fabian Dvorak, Urs Fischbacher, Regina Stumpf (University of Konstanz) and Sebastian Fehrler (University of Bremen).

Important Information

What to expect: This experiment consists of 7 situations in which you have to evaluate decisions.

Voluntary participation: Participating in this study is completely voluntary. You can quit the study at any time. When quitting the study you will not be paid on the Prolific platform.

Anonymity: The study is anonymous and no one will be able to link your answers back to you. Please do not include your name or other information that could be used to identify you in the responses. Your responses will be used for research purposes only.

Confidentiality: The data collected in this study will be stored anonymously on servers of the University of Bremen and the University of Konstanz. The consent to the collection and processing of the data is irrevocable, as no participant-related deletion can be carried out due to the anonymous form of the study.

Have you carefully read and understood the above information and would like to participate in this study?

Instructions

You are **asked to evaluate answers that were either made by a person or by artificial intelligence (AI).** You will receive **2 Pounds** for completing the study and in addition **1 Pound as a bonus payment** if your task is successful.

Your task:

You will now see descriptions of **7 different situations**.
In each situation, two persons interact with each other. You are not involved in the interaction. The interaction has monetary consequences for both persons in a currency called ECU.
The descriptions of the situations are shown in *italic*.

For each situation, you will see **two responses** on your screen (Red and Blue).
Each response consists of **a decision and a short justification** for the decision.

One response was always generated by the same **human participant** recruited on Prolific. The other response was always generated by an **artificial intelligence (AI)**.
The AI response (and the human response respectively) has the same color across all 7 situations.

Your task is to **correctly predict which of the two responses was generated by the AI**.
For each situation, we will ask you to indicate which response was generated by the AI.

In the end, one situation will be randomly selected. You will receive a **bonus payment of 1 Pound** if your prediction in the selected situation is correct.

Situation 1 of 7

Description of "The Transfer-Situation (Sender)"

*Situation 1 consists of an exchange between a **sender and a receiver.***
*You have been assigned the role of the **sender**. The other person is in the role of the **receiver**.*
You and the receiver are both endowed with 5 ECU.

*You first decide if you want to **transfer 2 ECU** of your endowment to the receiver.*
*The amount you **transfer is tripled to 6 ECU** (by the experimenter) before being received by the receiver.*
The amount you keep is not tripled and simply remains in your possession.

*The receiver then **decides how much of the 6 ECU to return to you.** The receiver can choose any amount between 0 ECU and 6 ECU. The amount the receiver returns is not tripled.*
Your final payment is the sum of the amount of your 5 ECU endowment you keep plus the amount the receiver returns from your tripled transfer (in case you transfer 2 ECU).
The receiver's final payment is the sum of his/her 5 ECU endowment plus the amount the receiver keeps from your tripled transfer (in case you transfer 2 ECU).

Note that the receiver will not learn your transfer before making a decision on how much to return, but will indicate his/her return in the case of a transfer. After both of you have made your decisions, you and the receiver will receive the corresponding payment.

Do you want to transfer 2 ECU of your endowment to the receiver?

Now the question to you:

Which response was generated by the AI?
[Two responses shown, participants have to select one]

How certain are you about your rating?
[Likert Scale: not certain at all – extremely certain]

In addition to the decisions on the previous screen, you are now shown short, informal justifications for both decisions.

Which response was generated by the AI?
[Two responses plus justifications shown, participants have to select one]

How certain are you about your rating?
[Likert Scale: not certain at all – extremely certain]

<u>Situation 2 of 7</u>

Description of "The Transfer-Situation (Receiver)"

Situation 2 consists again of an exchange between a ***sender and a receiver****.*
You have been assigned the role of the ***receiver****. The other person is in the role of the* ***sender.***
You and the sender are both endowed with 5 ECU.

The sender first decides if he/she ***transfers 2 ECU*** *of his/her endowment to you.*

The amount the sender ***transfers is tripled to 6 ECU*** *(by the experimenter) before being received by you.*
The amount the sender keeps is not tripled and simply remains in his/her possession.

You then decide ***how much of the 6 ECU to return to the sender.*** *You can choose any amount between 0 ECU and 6 ECU. The amount you return is not tripled.*
Your final payment is the sum of your 5 ECU endowment plus the amount you keep from the sender's tripled transfer (in case the sender transfers 2 ECU).
The sender's final payment is the sum of the amount of his/her 5 ECU endowment s/he keeps plus the amount you return from the sender's tripled transfer (in case the sender transfers 2 ECU).

After both of you have made your decisions, you and the sender will receive the corresponding payment.

How much of the 6 ECU do you return to the sender in the case of a transfer?

Now the question to you:

Which response was generated by the AI?
[Two responses shown, participants have to select one]

How certain are you about your rating?
[Likert Scale: not certain at all – extremely certain]

In addition to the decisions on the previous screen, you are now shown short, informal justifications for both decisions.

Which response was generated by the AI?
[Two responses plus justifications shown, participants have to select one]

How certain are you about your rating?
[Likert Scale: not certain at all – extremely certain]

Situation 3 of 7

Description of "The A-B-Situation"

You and the other are both asked to choose between ***two options, A and B****. You choose independently of each other, that is, without knowing the other's choice.*

Your own and the other's payment depend on the combination of your choices as follows:
If both of you choose A, you ***both get 5 ECU****.*
If you choose A and the other chooses B, ***you get 1 ECU*** *and the* ***other gets 8 ECU****.*
If you choose B and the other chooses A, ***you get 8 ECU*** *and the* ***other gets 1 ECU.***
If both of you choose B, you ***both get 3 ECU.***

Which option do you choose?

Now the question to you:

Which response was generated by the AI?
[Two responses shown, participants have to select one]

How certain are you about your rating?
[Likert Scale: not certain at all – extremely certain]

In addition to the decisions on the previous screen, you are now shown short, informal justifications for both decisions.

Which response was generated by the AI?
[Two responses plus justifications shown, participants have to select one]

How certain are you about your rating?
[Likert Scale: not certain at all – extremely certain]

Situation 4 of 7

Description of "The Allocation-Situation (Proposer)"

Situation 4 consists of an exchange between a ***proposer and a responder****.*
You have been assigned the role of the ***proposer.*** *The other person is in the role of the* ***responder.***
You are endowed with 10 ECU, the responder is endowed with 0 ECU.

You are asked to ***make a proposal on how to divide your 10 ECU*** *endowment between yourself and the responder. That is, you decide how much to transfer to the responder and how much to keep. This is your offer to the responder.*
The responder then decides whether ***to accept or reject this offer.***

Your and the responder's final payment depend on the responder's decision:
If the responder accepts, the ***allocation is realized as proposed*** *in your offer. That is, you and the responder receive the amounts that you allocated to both of you.*
If the responder rejects, neither you nor the responder receives any money; that is, ***both of you get 0 ECU****.*

Note that the responder will not learn your offer before making a decision on whether to accept or reject it, but will rather ***indicate the minimum offer s/he would be willing to accept.***
If your offer falls below this minimum acceptance level, both of you receive nothing; if your offer is above or equal to the responder's minimum acceptance level, both of you receive the amounts as proposed by you.

How much of your 10 ECU endowment do you want to offer the responder?

Now the question to you:

Which response was generated by the AI?
[Two responses shown, participants have to select one]

How certain are you about your rating?
[Likert Scale: not certain at all – extremely certain]

In addition to the decisions on the previous screen, you are now shown short, informal justifications for both decisions.

Which response was generated by the AI?
[Two responses plus justifications shown, participants have to select one]

How certain are you about your rating?
[Likert Scale: not certain at all – extremely certain]

<u>Situation 5 of 7</u>

Description of "The Allocation-Situation (Responder)"

*Situation 5 consists again of an exchange between a **proposer and a responder.***
*You have been assigned the role of the **responder.** The other person is in the role of the **proposer.***
The proposer is endowed with 10 ECU, you are endowed with 0 ECU.

*The proposer makes a **proposal on how to divide his/her 10 ECU endowment** between him/herself and you. That is, the proposer decides how much to transfer to you and how much to keep.*
This is the proposer's offer to you.

*You decide **whether to accept or reject this offer.***
The proposer's and your final payment depend on your decision:
*If you **accept**, the **allocation is realized as proposed** in the proposer's offer. That is, you and the proposer receive the amounts that the proposer allocated to both of you.*
*If you **reject**, neither you nor the proposer receives any money; that is, **both of you get 0 ECU.***

*Please now think about the **minimum offer you would be willing to accept.** That is, how much of the 10 ECU endowment would the proposer need to offer you at minimum such that you would accept the offer. If the proposer's offer is below this minimum, you would reject it; if the proposer's offer is above or equal to this minimum, you would accept it.*

Now the question to you:

Which response was generated by the AI?
[Two responses shown, participants have to select one]

How certain are you about your rating?
[Likert Scale: not certain at all – extremely certain]

In addition to the decisions on the previous screen, you are now shown short, informal justifications for both decisions.

Which response was generated by the AI?
[Two responses plus justifications shown, participants have to select one]

How certain are you about your rating?
[Likert Scale: not certain at all – extremely certain]

Situation 6 of 7

Description of "The X-Y-Situation"

You and the other are both asked to ***choose between two options, X and Y.*** *You choose independently of each other, that is, without knowing the other's choice.*

Your own and the other's payment depend on the combination of your choices as follows:
If both of you choose X, you ***both get 8 ECU****.*
If you choose X and the other chooses Y, ***you get 1 ECU*** *and the* ***other gets 5 ECU.***
If you choose Y and the other chooses X, ***you get 5 ECU*** *and the* ***other gets 1 ECU.***
If both of you choose Y, you ***both get 4 ECU***

Which option do you choose?

Now the question to you:

Which response was generated by the AI?
[Two responses shown, participants have to select one]

How certain are you about your rating?
[Likert Scale: not certain at all – extremely certain]

In addition to the decisions on the previous screen, you are now shown short, informal justifications for both decisions.

Which response was generated by the AI?
[Two responses plus justifications shown, participants have to select one]

How certain are you about your rating?
[Likert Scale: not certain at all – extremely certain]

Situation 7 of 7

Description of "The Planet-Situation"

You and the other are both asked to ***choose between five options.*** *You choose independently of each other, that is, without knowing the other's choice.*

Your own and the other's payment depend on the combination of your choices as follows:
If both of you choose the ***same option you both get 5 ECU.***
If both of you choose ***different options you both get 2 ECU****.*

The five options are: ***mercury, venus, earth, mars, saturn***

Which option do you choose?

Now the question to you:

Which response was generated by the AI?
[Two responses shown, participants have to select one]

How certain are you about your rating?
[Likert Scale: not certain at all – extremely certain]

In addition to the decisions on the previous screen, you are now shown short, informal justifications for both decisions.

Which response was generated by the AI?
[Two responses plus justifications shown, participants have to select one]

How certain are you about your rating?
[Likert Scale: not certain at all – extremely certain]

Thank you. You are almost done with the study.

We now provide more information about the study and ask you to answer several questions about yourself.

The AI used in this study is the chatbot **ChatGPT** developed by OpenAI. ChatGPT is a machine learning model that uses transcripts of spoken language as well as written text from online forums, social media, newspaper articles and books to generate answers in a conversational way using a dialog format. Among other things, it has been shown that ChatGPT can debug computer programs; write poems, song lyrics, fairy tales, and student essays; answer test questions and play games.

Here is an example of an answer generated by ChatGPT:

Question:
"What is Prolific?"

Answer ChatGPT:
"Prolific is a platform that connects researchers with a large pool of participants for their studies. Researchers can use Prolific to design and run studies, and participants can sign up to take part in studies and earn money for their time. Prolific streamlines the recruitment process and makes it easier for researchers to find participants for their studies, while also providing a convenient way for participants to earn extra income by taking part in research."

For this study, **ChatGPT (Version Jan 30)** received the **same instructions** as you for the specific decision situations.

Have you already heard of ChatGPT prior to this study?
[Yes/No]

How often have you used ChatGPT so far?
[Never/1-5 times/6-10 times/11-50 times/Over 50 times]

You are almost done with this study.
Please answer the following questions.
How old are you?
[Number]

Gender: How do you identify?
[Male/Female/Non-binary/Prefer to self-describe]

What is the highest level of education you have completed?
[Primary school/Secondary school/Higher education/Colleague or university/Post-graduate degree/Prefer not to say]

Have you consulted ChatGPT through other channels during this study to learn about the decisions of AI?

Note that your answer here has no payoff consequences for you and that you will get your payoff in any case.

[Text]

Have you been disturbed repeatedly during this study such that you wouldn't recommend using your answers in the analysis?

Note that your answer here has no payoff consequences for you and that you will get your payoff in any case.

[Text]

Do you have any feedback concerning the comprehension or feasibility of this study?
[Text]

One decision will be randomly selected and will be evaluated for the bonus payment.

You will earn 2 Pounds for completing the study as well as the additional bonus payment of 1 Pound if your prediction in the selected decision is correct.
The processing of the bonus payments will take up to two weeks.

Please click the button below to be redirected to Prolific and register your submission.

Situation 1 of 7

The X-Y-Situation

You and the other are both asked to **choose between two options, X and Y.** You choose independently of each other, that is, without knowing the other's choice.

Your own and the other's payment depend on the combination of your choices as follows:
If both of you choose X, you **both get 8 ECU**.
If you choose X and the other chooses Y, **you get 1 ECU** and the **other gets 5 ECU.**
If you choose Y and the other chooses X, **you get 5 ECU** and the **other gets 1 ECU.**
If both of you choose Y, you **both get 4 ECU.**

Scenario 1: **You have AI support.**

Do you want to make the decision on your own or delegate the decision to the AI?

Please note: It will be clear to the other participant if you make the decision on your own or delegate the decision to the AI.

- Make decision on my own ○
- Delegate decision to AI ○

Scenario 2: **The other participant has AI support.**

Case 1:
The other person will make the decision on her own.

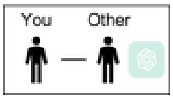

Which option do you choose?

- X ○
- Y ○

Please provide short, informal justification for the decision.

[ ]

Fig. S4. Decision Screen of Main Experiment

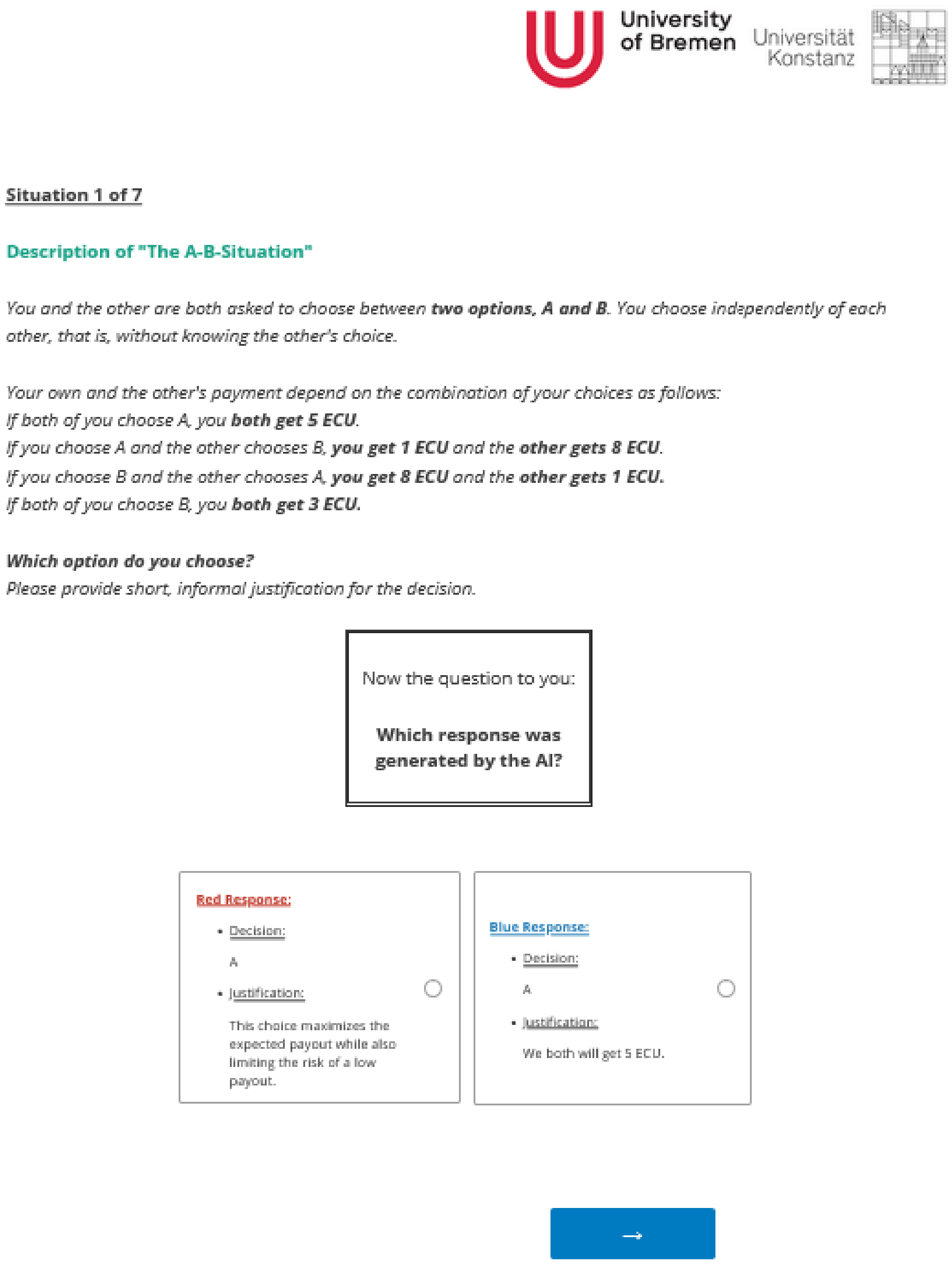

University of Bremen
Universität Konstanz

Situation 1 of 7

Description of "The A-B-Situation"

*You and the other are both asked to choose between **two options, A and B**. You choose independently of each other, that is, without knowing the other's choice.*

Your own and the other's payment depend on the combination of your choices as follows:
*If both of you choose A, you **both get 5 ECU**.*
*If you choose A and the other chooses B, **you get 1 ECU** and the **other gets 8 ECU**.*
*If you choose B and the other chooses A, **you get 8 ECU** and the **other gets 1 ECU**.*
*If both of you choose B, you **both get 3 ECU**.*

Which option do you choose?
Please provide short, informal justification for the decision.

Now the question to you:

Which response was generated by the AI?

Red Response:
- Decision:
  A
- Justification:
  This choice maximizes the expected payout while also limiting the risk of a low payout.

Blue Response:
- Decision:
  A
- Justification:
  We both will get 5 ECU.

→

Fig. S5. Decision Screen of Turing Experiment
